# The Eradication of Venereal Disease

**Published:** 1916-12-16

**Authors:** 


*To the Editor of* The Hospital.

### The Eradication of Venereal Disease.

*To the Editor of* The Hospital.

Sir,—Public attention has at last been aroused to the national danger involved in the prevalence of venereal diseases.

We congratulate the Government on the measures taken to secure that throughout the country treatment centres will shortly be open where all sufferers may obtain free treatment under reasonable and convenient conditions. We believe that such facilities, combined with public enlightenment, afford the best hope of preventing the spread of infection. We concur in the view of the Royal Commission as to the danger of frustrating these efforts by the addition of any kind of compulsory measures, which always tend to drive the disease underground.

Suggestions are sometimes made for dealing with this scourge by restrictions imposed only on women. Such efforts always have been and always must be futile. While men complain that they are infected by some woman, each woman may, with equal certainty, affirm that she was infected by some man; and it is usually by men that infection is carried to unsuspecting wives and innocent children.

We urge the Government and Parliament and all persons interested in the subject to apply the following tests to every proposal dealing with this great problem :—

Firstly, will it tend to strengthen or to weaken that sense of individual responsibility in relation to sexual conduct which is the strongest and most effectual bulwark against the spread of disease?

And, secondly, can it and will it in actual practice be applied with impartial justice to both sexes and to all classes?

Only by measures which can satisfactorily meet these tests will the eradication of venereal diseases be brought about.

Signed by :—

Councillor Margaret Ashton, Manchester.

Lady Frances Balfour.

Lady Barrett, M.D., M.S.

Mrs. Barton (Women's Co-operative Guild).

Miss Aldrich Blake, M.S., M.D. (Lond.).

Mrs. Bramwell Booth.

Miss Annie Leigh Browne, Hon. Sec. Women's Local Government Society.

Mrs. Burrows (Sheffield).

Mrs. Sophie Bryant, D.Sc.

Lady Frederick Cavendish.

Mrs. George Cadbury.

Miss Janet Case (London).

Lady Cohen (Hythe).

Miss Clara Collet, M.A.

Countess of Chichester.

Mrs. Crawford, Hon. Sec. Catholic Social Guild.

Mrs. John Clay, Cambridge.

Mrs. Creighton.

Miss Llewelyn Davies, Women's Co-operative Guild.

Mrs. Katherine Dixon.

Lady Emmott.

Mrs. Henry Fawcett.

Lady Forbes Robertson.

Mrs. Edwin Gray, York.

Mrs. Ogilvie Gordon, D.Sc., Ph.D.

Mrs. Gotto.

Mrs. Gow, Westminster.

Mrs. Hugh Price Hughes, London.

Mrs. Kempthorne, Lichfield.

Hon. Emily Kinnaird.

Mrs. Pethick Lawrence.

Miss Blanche Leppington.

Mrs. Archibald Little.

Miss Mary Macarthur.

Mrs. How Martyn.

Hon. Lily Montagu.

Miss Marion Phillips, D.Sc.

Dr. A. Renshaw, Manchester.

Lady Laura Ridding.

Miss Maude Royden.

Mrs. Walter Runciman.

Mrs. Scharlieb, M.D., M.S.

Mrs. Selbie (Oxford).

Countess of Selbourne.

Dr. Amy Sheppard.

Lady Spicer.

Dr. Jane Walker.

Dr. Helen Webb.

Dr. Helen Wilson

Mrs. Henry J. Wilson (Sheffield).

Mrs. Wilberforce (Mothers' Union, President of Central).

[We print this letter as received, and are in agreement with much of it, but we cannot help feeling that the signatories are spoiling a strong case by injudicious advocacy. The statement that "such efforts always have been and always must be futile" is a sentence in point. The first half of it is of very doubtful accuracy, and the second half is an expression of opinion which can only be described as unscientific and unworthy of its distinguished sponsors. Again, the two "tests" which it is proposed to apply to proposals for reducing venereal diseases seem to us much more debatable and less effective than the single test we would ourselves apply : which is, "Will this proposal tend to diminish the prevalence and the physical ill-effects of venereal disease?" The letter which Dr. Helen Wilson sends us takes for granted certain views which may be correct enough, but are not strictly proven; and to that extent it weakens the cause which she and her fellow-signatories, of course, have just as much at heart as has the Editor of The Hospital.]

